# Cytokines from Macrophages Activated by Spike S1 of SARS-CoV-2 Cause eNOS/Arginase Imbalance in Endothelial Cells

**DOI:** 10.3390/ijms26125916

**Published:** 2025-06-19

**Authors:** Giulia Recchia Luciani, Rossana Visigalli, Valeria Dall’Asta, Bianca Maria Rotoli, Amelia Barilli

**Affiliations:** Laboratory of General Pathology, Department of Medicine and Surgery, University of Parma, 43125 Parma, Italy; giulia.recchialuciani@unipr.it (G.R.L.); rossana.visigalli@unipr.it (R.V.); biancamaria.rotoli@unipr.it (B.M.R.); amelia.barilli@unipr.it (A.B.)

**Keywords:** arginine, arginase, cytokines, endothelial dysfunction, eNOS, macrophages, TNFα

## Abstract

Multiple lines of evidence suggest that endothelial dysfunction is a key player in the pathogenesis of COVID-19, with cytokine storm as one of the main primary causes. Among the mechanisms underlying endothelial damage, clinical findings identify alterations in arginine metabolism, as patients with severe COVID-19 exhibit lower levels of nitric oxide synthase (eNOS) and upregulated arginase. In this study, we investigated, in human endothelial cells (HUVECs), the effect of conditioned medium from macrophages activated with SARS-CoV-2 Spike protein (CM_S1) on arginine metabolism. The results indicate that CM_S1 causes a marked decrease in eNOS and an increase in arginase, along with a greater intracellular arginine content and the induction of the CAT2 transporter. These effects are ascribable to the inflammatory mediators released by macrophages in CM_S1, mainly TNFα and IL-1β. Since infliximab, an antibody targeting TNFα, and baricitinib, an inhibitor of the JAK/STAT pathway, correct the observed imbalance between eNOS and arginase, our findings suggest the potential efficacy of a combined therapy to counteract endothelial dysfunction in COVID-19.

## 1. Introduction

Although COVID-19 mainly manifests as a viral pneumonia resulting in acute respiratory failure, it is nowadays recognized as a multiorgan disease in which endothelial dysfunction (ED) plays a prominent role [1]. Multiple lines of evidence suggest, indeed, that endothelial activation is an early hallmark of damage in patients with COVID-19 [2] and points to ED as a key player in the pathogenesis of the early stages of the disease, as well as in the progression to severe late complications [3], such as acute respiratory distress syndrome (ARDS), myocardial damage, systemic vasculitis, or thrombotic complications [4].

Endothelial dysfunction in COVID-19 is ascribable to the direct effects of SARS-CoV-2 infection on endothelial cells but also to the exacerbation of the immune response secondary to viral infection, which leads to a dysregulated release of pro-inflammatory mediators [5]. It has been ascertained, indeed, that the hyperproduction of inflammatory cytokines (the so-called “cytokine storm”) associates with COVID-19 severity and endothelial injury [3]. In particular, TNFα, IFNγ, IL-6, IL-1β, GM-CSF, and G-CSF have been identified as the main mediators responsible for endothelial activation and dysfunction following SARS-CoV-2 infection [6]. Moreover, huge amounts of inflammatory mediators in the lungs lead to a severe impairment of the endothelium, resulting in increased vascular permeability, which is the main cause of pulmonary edema in ARDS [7].

Mounting evidence also indicates that SARS-CoV-2 infection deeply affects arginine metabolism and suggests that these modifications, in patients with COVID-19, are associated with immune and vascular dysfunction [8,9]. Arginine is a semi-essential amino acid metabolized by nitric oxide synthase (NOS) to citrulline and nitric oxide (NO) or alternatively by arginase to urea and ornithine. Endothelial cells express the constitutive isoform of NOS, the endothelial nitric oxide synthase (eNOS) [10], and the synthesized NO represents the key mechanism for maintaining endothelial homeostasis through regulating vascular tone and anti-thrombotic action [11]. Consistently, the loss of endothelium-derived NO has been shown to result in serious cardiovascular abnormalities [12,13]. As for arginase, two distinct isoforms of the enzyme have been detected in endothelial cells, namely the cytosolic arginase I (Arg1) and mitochondrial arginase II (Arg2). Arg1 is mostly expressed in the liver, where it is involved in urea synthesis; Arg2 is, instead, widely expressed in several tissues, where, by promoting the synthesis of L-ornithine, a precursor of polyamines and proline, it regulates collagen synthesis, tissue repair, and proliferation [14,15,16].

The maintenance of NOS/arginase balance is fundamental for the correct preservation of endothelial function. Interestingly, COVID-19 severe patients show the inhibition of endothelial nitric oxide synthase (eNOS), leading to a concurrent deficiency in nitric oxide (NO) production and an up-regulation of arginase that, instead, favors the production of ornithine and its metabolites from arginine over the synthesis of NO [17,18]. Consistently, SARS-CoV-2 infection leads to a decrease in NO production through mechanisms including the direct infection of endothelial cells and an indirect effect due to the cytokine storm [19]. These evidence suggest that a reduction in NO bioavailability can play a role in ED associated with COVID-19. Since NO also possesses antiviral properties, the therapeutic action of NO administration to COVID-19 patients has been evaluated [20,21,22].

As arginine is the common substrate of eNOS and arginase, the supply of adequate amounts of this amino acid is a prerequisite for the correct maintenance of NOS/arginase balance. In human endothelial cells, arginine availability mostly depends on the activity of two trans-membrane transport systems: System y+, membrane potential-dependent, specific for cationic amino acids, and System y+L, an exchanger that also accepts neutral amino acids, such as leucine [23]. System y+ consists of the monomeric cationic amino acid transporters CAT1 and CAT2 (encoded by SLC7A1 and SLC7A2 genes), while System y+L activity is due to a heterodimeric amino acid transporter formed by 4F2hc/CD98 and one of two alternative light subunits, y+LAT1 or y+LAT2 (encoded by SLC7A7 and SLC7A6 genes, respectively).

The precise relation between cytokine-induced endothelial dysfunction and the alteration of arginine metabolism in COVID-19 remains to be clarified. In a recent contribution, we demonstrated that inflammatory mediators released by macrophages activated by the Spike S1 protein of SARS-CoV-2 impair endothelial cell viability and proliferation [24]. Here, to further explore the mechanisms responsible for vascular complications upon SARS-CoV-2 infection, we focused on the role of cytokines in the modulation of arginine metabolism in endothelial cells. To this end, the expression of eNOS/arginase, the intracellular arginine content, and NO production have been addressed in human umbilical endothelial cells (HUVECs), along with the underlying molecular pathways; the beneficial effect of the drugs baricitinib and infliximab has also been investigated.

## 2. Results

**Figure 1:**
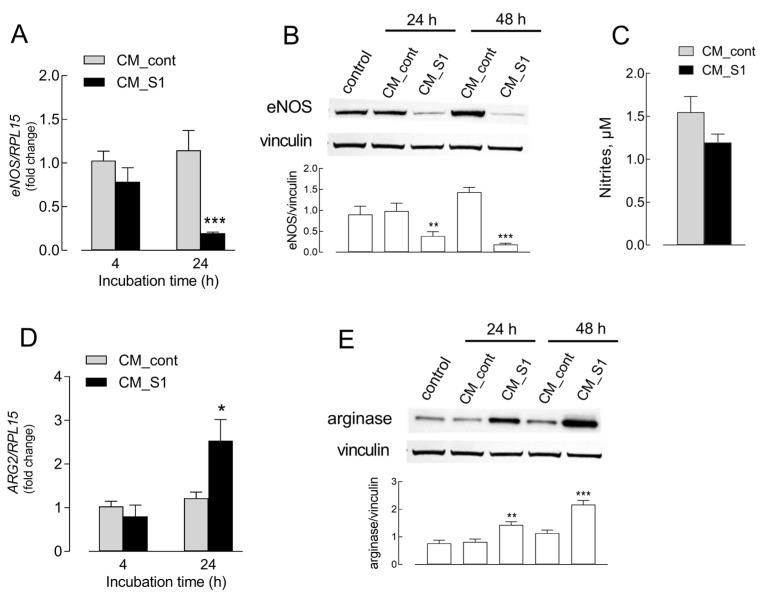
HUVECs were incubated in conditioned medium (CM) obtained from monocyte-derived macrophages untreated (CM_cont) or treated for 24 h with 5 nM S1 (CM_S1); for control, cells were maintained in RPMI1640 medium. After 4 and 24 h, the mRNA levels of NOS3 (**A**) and ARG2 (**D**) were assessed with RT-qPCR and shown as a fold change in control (=1). Bars are means ± SEM of four experiments performed in duplicate. * *p* < 0.05, *** *p* < 0.001 vs. control with Student’s *t* test. eNOS (**B**) and arginase (**E**) protein expression was determined with Western Blot analysis, as detailed in the Methods section. Representative blots are shown, along with the mean ± SEM of the densitometry analysis in three different experiments. ** *p* < 0.01, *** *p* < 0.001 vs. control cells with One-way ANOVA. (**C**). After 48 h of incubation in the presence of CM_S1, the amount of nitrites in the culture medium was assessed (see Methods). Bars are means ± SEM of three independent experiments.

In order to investigate the involvement of arginine metabolic pathways in COVID-19-associated endothelial damage, we first evaluated the immune-mediated effects of Spike S1 protein of SARS-CoV-2 on the expression of both nitric oxide synthase (NOS3/eNOS) and ARG2/arginase enzymes in endothelial cells in vitro. To this aim, HUVECs were incubated for 4 and 24 h in the presence of a conditioned medium obtained from untreated macrophages (CM_cont) or from macrophages treated with S1 protein (CM_S1). As shown in Figure 1A, this treatment caused a reduction in NOS3 mRNA levels, particularly impressive after 24 h, when the decrease reached statistical significance. The same effect was even more evident at the protein level (Figure 1B): only a faint band corresponding to eNOS was, indeed, detectable after 24 or 48 h in the presence of CM_S1. Consistently, a modest, not significant decrease in nitrites, stable derivatives of nitric oxide, in the culture medium was observed after 48 h of incubation with CM_S1 (Figure 1C). In parallel, an increase in arginase mRNA levels was detected in cells treated for 24 h with CM_S1 (Figure 1D); a progressive increase in arginase protein was also observed (Figure 1E).

**Figure 2:**
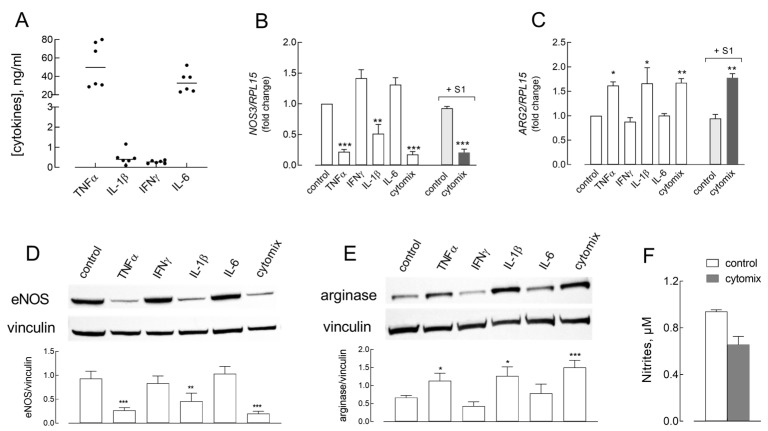
(**A**) The amount of the indicated cytokines in CM_S1 was determined as described in the Methods section. (**B**–**E**) HUVECs were incubated in RPMI1640 medium, in the absence (control) or in the presence of the indicated cytokines, employed alone or combined (cytomix). Where indicated, 5 nM of Spike S1 was added. The mRNA expression of NOS3 (**B**) and ARG2 (**C**) was measured after 24 h of incubation using RT-qPCR. Data are expressed as a fold change in the expression in control cells (=1). Bars are means ± SEM of four experiments, each performed in duplicate. * *p* < 0.05, ** *p* < 0.01, and *** *p* < 0.001 vs. control, untreated cells with One-way ANOVA. After 48 h, the expression of eNOS (**D**) and arginase (**E**) proteins was assessed with Western Blot (see Methods). Representative blots are shown, along with mean ± SEM of the densitometry analysis in three different experiments. * *p* < 0.05, ** *p* < 0.01, and *** *p* < 0.001 vs. control cells with One-way ANOVA. (**F**). After 48 h of treatment in the presence of cytomix, the amount of nitrites in the culture medium was assessed (see Methods). Bars are means ± SEM of three independent experiments.

We have previously reported that the conditioned medium from S1-treated human macrophages contains many inflammatory mediators, including the cytokines TNFα, IL-1β, IL-6, and IFNγ [25,26]. Here, we determined the amount of these cytokines in CM_S1 obtained from six healthy donors, which were pooled and used for the experiments; the results are shown in Figure 2A. To verify the involvement of these mediators in the effects caused by CM_S1, we next addressed the effects of 50 ng/mL of TNFα or IL-6 and of 5 ng/mL of IL-1β or IFNγ, used alone or combined (cytomix), on the expression of eNOS and arginase. The results, presented in Figure 2B–E, demonstrate that both IL-1β and, even more markedly, TNFα caused a strong, significant reduction in eNOS expression, both as mRNA, after 24 h of treatment (Figure 2B), and protein, after 48 h of treatment (Figure 2D). Conversely, neither IFNγ nor IL-6 had an effect on eNOS expression. As expected, a drop in eNOS level was also observed upon incubation with cytomix, which contains all four cytokines; under this condition, the reduction in protein expression was comparable to that induced by TNFα or IL-1β, sustaining a prominent contribution of these cytokines to the effect observed. In the presence of cytomix, a modest reduction in nitrite production was also observed (Figure 2F). As for ARG2 expression, mRNA levels were significantly increased by both TNFα and IL-1β and by cytomix, but not by IFNγ and IL-6 (Figure 2C). The same experimental conditions caused a significant increase also at the protein level (Figure 2E). Moreover, the addition of Spike S1 protein to the incubation medium did not affect the expression of NOS3 (Figure 2B) nor ARG2 (Figure 2C) under basal conditions and did not even strengthen the effect of cytomix, excluding a direct role of the viral protein on endothelial cells.

**Figure 3:**
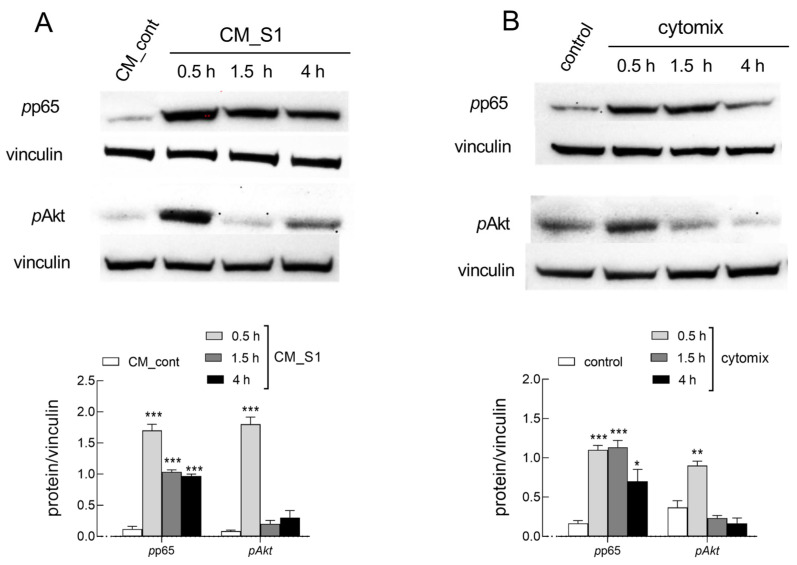
HUVECs were incubated in CM_S1 (**A**) or in the presence of cytomix (**B**). After the indicated times, the expression of the indicated proteins was assessed with Western Blot (see Methods). Representative blots are shown, along with mean ± SEM of the densitometry analysis in three different experiments. * *p* < 0.05, ** *p* < 0.01, and *** *p* < 0.001 vs. control cells with One-way ANOVA.

In light of the role played by inflammatory mediators, mostly TNFα or IL-1β, in the effects described, the activation of the signaling pathways of protein kinase B (PKB/Akt) and NF-κB, among the several known downstream targets of the cytokines [27,28], has been evaluated in CM_S1- and cytomix-treated cells. As shown in Figure 3, a transient phosphorylation of Akt in Thr308 and a more prolonged phosphorylation of p65, a subunit of NF-κB, have been observed under both experimental conditions, indicating the activation of the two molecular pathways.

**Figure 4:**
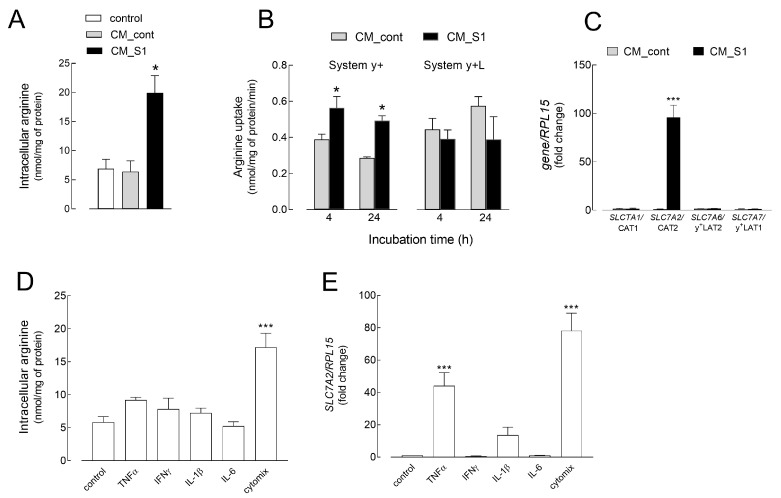
(**A**–**C**) HUVECs were treated as in Figure 1. (**A**) After 48 h, the intracellular content of arginine was determined as described in the Methods section. Bars are the means ± SEM of four experiments. * *p* < 0.05 vs. CM_cont with Student’s *t* test. (**B**) At the times indicated, arginine uptake was determined by measuring the activity of systems y+ and y+L, as described in the Methods section. Data are the mean ± SEM of three experiments, each performed in quadruplicate. * *p* < 0.05 vs. CM_cont with Student’s *t* test. (**C**) After 24 h, the expression of arginine transporters was measured using RT-qPCR and expressed as a fold change in cells maintained in RPMI1640 medium (control = 1). Bars are means ± SEM of three experiments, each performed in duplicate. *** *p* < 0.001 vs. control with Student’s *t* test. (**D**,**E**) HUVECs were incubated in RPMI1640 medium, either in the absence (control) or in the presence of the indicated cytokines, employed alone or combined (cytomix). (**D**) After 48 h, the arginine intracellular content was determined as described in the Methods section. Bars are means ± SEM of four experiments. *** *p* < 0.001 vs. control cells with One-way ANOVA. (**E**) SLC7A2/CAT2 mRNA expression was measured after 24 h of incubation by means of RT-qPCR. Data are expressed as a fold change in gene levels in control (=1). Bars are the means ± SEM of four experiments, each performed in duplicate. *** *p* < 0.001 vs. control, untreated cells with One-way ANOVA.

To evaluate whether the observed changes in arginine metabolic pathways were associated with alterations of the availability of the amino acid, the intracellular content of arginine was measured after treatment with CM_S1 or with the cytokines (Figure 4). As shown in Figure 4A, arginine levels, comparable in control and CM_cont-treated cells, significantly increased after 48 h of incubation with CM_S1, when they were more than doubled. This increase prompted us to measure arginine influx by evaluating the contribution of transport Systems y+ and y+L (Figure 4B). A significant increase in System y+ activity occurred in cells treated with CM_S1 at both 4 and 24 h, while no change was observed for System y+L at any time of incubation. Consistently, the expression of the mRNA for System y+L-related transporters, SLC7A6/y+LAT2 and SLC7A7/y+LAT1, was not modified in the presence of CM_S1. As for System y+, no change was observed in the expression of SLC7A1/CAT1, while the expression of SLC7A2/CAT2 was greatly stimulated. As shown in Figure 4D, the intracellular levels of arginine were also slightly increased by the incubation with TNFα, although the increase reached statistical significance only in the presence of cytomix, a condition where arginine content almost tripled, reaching values comparable to that of CM_S1. In line with these findings, a significant induction of CAT2 expression was evident after 24 h of incubation with TNFα or cytomix, supporting a role for this cytokine in increasing the intracellular arginine concentration (Figure 4E).

**Figure 5:**
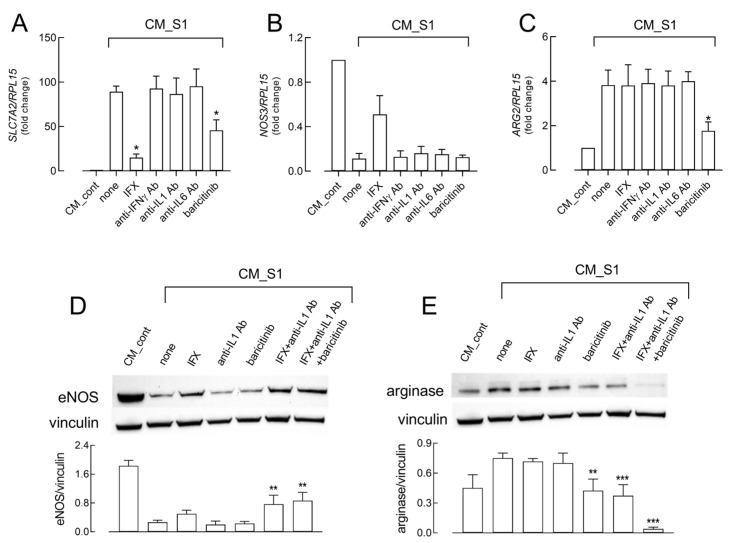
HUVECs were incubated with CM_cont or CM_S1; the drugs infliximab (IFX) or baricitinib and the antibodies against the indicated cytokines were added to CM_S1, as indicated. After 24 h, the mRNA levels of SLC7A2 (**A**), NOS3 (**B**), and ARG2 (**C**) were measured by means of RT-qPCR and expressed as a fold change in CM_cont (=1). Bars are means ± SEM of three experiments, each performed in duplicate. * *p* < 0.05 vs. none with One-way ANOVA. After 48 h, the expression of eNOS (**D**) and arginase (**E**) proteins were assessed through Western Blot analysis (see Methods). Representative blots are shown, along with the mean ± SEM of the densitometry analysis of three different experiments. ** *p* < 0.01, *** *p* < 0.001 vs. none with One-way ANOVA.

To confirm this hypothesis, as well as to ascertain the contribution of each cytokine to the observed effects, specific neutralizing antibodies were added to CM_S1; in addition, the JAK/STAT inhibitor baricitinib, a drug approved for COVID-19 treatment, was also tested (Figure 5). As shown in Figure 5A, antibodies targeting IFNγ, IL-1β, and IL-6 had no effect on the induction of the SLC7A2/CAT2 transporter by CM_S1, while the presence of infliximab (IFX), a monoclonal antibody anti-TNFα, massively prevented it, confirming the prominent role of this cytokine in this issue. The addition of baricitinib limited the CM_S1-dependent increase in the mRNA for the transporter, suggesting that other mediators in conditioned medium are involved in the stimulation of arginine uptake. The same antibodies were also tested on the expression of eNOS and arginase. As shown in Figure 5B, only IFX limited the decrease in NOS3 mRNA induced by the treatment with CM_S1, while all the other inhibitors, including baricitinib, had no effect. This latter drug, however, was the only one able to partially prevent the induction of arginase mRNA (Figure 5C). The effective inhibitors were then further tested at the protein level, both alone and combined. The levels of eNOS, massively reduced by CM_S1, were partially restored only when infliximab was present in the incubation medium, either alone or more markedly in combination with anti-IL-1β antibody and/or baricitinib, which, per se, had no effect. As far as the expression of arginase is concerned, the combination of infliximab and anti-IL-1β antibody prevented protein induction by CM_S1 to the same extent as baricitinib, while the concomitant presence of the three inhibitors massively decreased arginase expression to levels even lower than in CM_cont-treated cells.

## 3. Discussion

Endothelial dysfunction (ED) is supposed to exert a pivotal role in the onset of COVID-19-associated complications, mainly respiratory, thrombotic, and myocardial manifestations [11]. Consistently, ED-related comorbidities such as hypertension, diabetes, obesity, and cardiovascular disease are known to increase the risk of severe COVID-19 [29].

Endothelial injury in COVID-19 is nowadays ascribed to both a direct cytopathic effect of SARS-CoV-2 on endothelial cells and to the uncontrolled release of cytokines and other inflammatory mediators secondary to the viral infection [30,31]. To this concern, we have recently demonstrated that the supernatants from Spike-activated macrophages (CM_S1) induce an inflammatory phenotype in human endothelial cells [32] and exert both cytostatic and cytopathic effects [24]. We also provided evidence ascribing a prominent role in the observed effects to the cytokines present in the conditioned medium; in particular, among the different mediators released by activated macrophages in vitro, IFNγ, TNFα, and IL-1β are supposed to be responsible, at least in part, for the attenuation of endothelial growth and the induction of cell toxicity.

Here, we demonstrate that the same conditioned medium also causes an almost complete suppression of NOS3/eNOS levels with a concomitant decrease in NO production, paralleled by an increase in arginase expression. These in vitro findings are consistent with clinical studies indicating that arginine metabolism is altered in COVID-19: a recent study found that COVID-19 patients with ARDS exhibit lower levels of soluble eNOS compared to COVID-19 non-ARDS patients [18]; the activity of arginase has been reported, instead, to be upregulated in patients with COVID-19, where it favors the production of ornithine and its metabolites from arginine over the synthesis of NO [17,33,34]. In line with the observed increase in arginase activity, circulating plasma levels of arginine have been described to progressively decrease as the severity of the disease increases [35,36,37,38]. Since the maintenance of NOS/arginase balance is fundamental for the correct preservation of endothelial function, our results demonstrating an immune-mediated loss of eNOS and induction of arginase suggest that this impairment may have a role in the onset of endothelial dysfunction associated with COVID-19. In line with these findings, abnormal arginase activity has been shown to contribute to the initiation and progression of a variety of cardiovascular diseases, likely due to the induction of endothelial senescence because of eNOS-uncoupling and increased oxidative stress [39]. Whether these events also intervene in COVID-19-associated ED remains to be ascertained, even if our findings exclude an excessive consumption of arginine as a possible cause for the uncoupling of eNOS under these conditions.

According to our results, these alterations of arginine metabolic pathways are ascribable to inflammatory mediators released by Spike-activated macrophages, mainly the cytokines TNFα and IL-1β. These findings are consistent with evidence in the literature from our group and others, showing that eNOS is profoundly down-regulated in the presence of proinflammatory cytokines, in particular TNFα [23,40,41,42] and IL-1β [43], as well as by pathophysiological stimuli such as hypoxia [44]. Similarly, arginase expression is known to be upregulated in endothelial cells by several stimuli, such as TNFα, lipopolysaccharide, oxidized low-density lipoproteins, peroxynitrites, hypoxia, angiotensin II, and reactive oxygen species [45]. In our hands, both TNFα and IL-1β are able to abolish the expression of eNOS, likely through independent pathways. Indeed, while the inhibition of TNFα alone by infliximab (IFX) partially restores protein levels, it is the concomitant presence of this drug and anti-IL1β antibody that almost completely prevents protein loss. Similarly, the treatment with either TNFα or IL-1β causes a significant increase in arginase expression, and only the simultaneous inhibition of both cytokines prevents protein induction; in this case, however, the JAK/STAT inhibitor baricitinib, already effective when employed alone, further diminishes arginase expression when combined with IFX and anti-IL1β antibody. Under this latter condition, the protein level is even lower than in CM_cont-treated endothelial cells, suggesting that this conditioned medium contains mediators released by control macrophages, able to induce arginase expression in HUVECs with a JAK/STAT-dependent mechanism.

Under our experimental conditions, the observed changes in eNOS and arginase are associated with an increase in the content of intracellular arginine. Since arginase competes with eNOS for arginine, elevated arginase levels are expected to deplete arginine availability; conversely, in our hands, higher intracellular levels of arginine were unexpectedly measured in CM_S1-treated endothelial cells, despite the significant induction of arginase. Based on these results, we can hypothesize that arginine intracellular availability and metabolism in endothelial cells are independently modulated by the cytokines present in the conditioned medium. Indeed, while the impairment of the eNOS/arginase balance is completely ascribable to the sole TNFα or IL-1β, arginine intracellular content markedly increases in the presence of cytomix, where a pivotal role is likely played by the increased uptake of the amino acid through System y+. In particular, a huge induction of the CAT2 transporter is observed in cytomix-treated cells, with a prominent contribution by TNFα. Indeed, we show here that TNFα but not IL-1β causes a massive induction of CAT2 expression, in line with our previous results demonstrating that TNFα but not IL-1β stimulates arginine transport in HUVECs [23]; moreover, the treatment with IFX is sufficient to completely prevent the induction of CAT2.

The hypothesis of an independent modulation of arginine uptake and metabolism by CM_S1 is in contrast with the many lines of evidence obtained in non-endothelial cells, mainly murine macrophages, where high amounts of NO are shown to be produced in response to cytokines or LPS through an increased uptake of arginine by CAT transporters [46,47,48]; in those contributions, however, NO production was due to the activation of the inducible isoform of NOS (iNOS/NOS2), rather than eNOS. Our results are, instead, consistent with findings obtained in HUVECs upon combined incubation with TNFα and the mTOR inhibitor rapamycin [41]. There, treated cells, although expressing higher levels of CAT2 and showing increased intracellular arginine concentration, synthesized less NO than control cells, due to the inhibition of eNOS expression.

Lastly, in our study, the pathways involving the activation of protein kinase B (PKB/Akt) and NF-κB represent signaling targets of both CM_S1 and cytomix treatments, as expected, as downstream events in the cascade triggered by cytokines. Although activated Akt is pivotal for eNOS activation [49], our findings demonstrate a reduction in NO production associated with a significant down-regulation of eNOS at both the transcriptional and protein levels, despite the rapid, transient phosphorylation of Akt. Whether the activation of Akt or NF-κB has a role in the transcriptional regulation, mRNA stability or post-translational modification of eNOS remains to be clarified.

Our findings gain particular relevance when considering the therapeutic potential of the two inhibitors, baricitinib and IFX. Baricitinib, approved by the FDA for the treatment of hospitalized patients requiring supplemental oxygen [50], proved effective in reducing mortality when used in addition to the current standard of care [51,52]. Conversely, the use of infliximab in the therapy of COVID-19 is still controversial, with some studies demonstrating significant benefits compared to standard of care in moderate to severe hospitalized COVID-19 patients [53,54,55] and others excluding the efficacy of this drug to ameliorate the clinical condition [56,57]. In our hands, both drugs proved effective in the correction of arginine metabolic derangement induced in endothelial cells by cytokines released by spike-activated macrophages. In particular, infliximab but not baricitinib was able to prevent eNOS decrease; vice versa, baricitinib limited arginase induction.

## 4. Materials and Methods

### 4.1. Cell Culture and Experimental Treatments

Human monocyte-derived macrophages (MDMs) were obtained as already described from monocytes isolated from the buffy coats of healthy donors provided by the Immunohematology and Transfusion Unit of the Azienda Ospedaliero-Universitaria of Parma [19]. The study protocol was approved by the local ethics committee (460/2021/TESS/UNIPR) and conducted according to the Declaration of Helsinki (1964). MDMs were generated with a 5-day incubation of adherent monocytes in RPMI1640 medium plus 10% fetal bovine serum and 50 ng/mL of Granulocyte Macrophage-Colony-Stimulating Factor (GM-CSF, RELIATech by Vinci-Biochem, Firenze, Italy). For the experiments, macrophages were incubated with or without 5 nM of the S1 subunit of the SARS-CoV-2 spike recombinant protein (ARG70218; Arigo Biolaboratories, Zhubey City, Taiwan,). Before the treatment, Spike S1 was pre-mixed with 2 µg/mL of Polymyxin B to eliminate possible contaminating lipopolysaccharides. Conditioned media from six different donors were collected after 24 h from control (CM_cont) and S1-treated MDMs (CM_S1) and pooled for further use.

Human Umbilical Vein Endothelial Cells (HUVECs) by Thermo Fisher Scientific (Monza, Italy) were cultured in Human Large Vessel Endothelial Basal Medium, added with Large Vessel Endothelial Supplement (LVES). Cells were used between one and six passages. For experiments, 5 × 10^4^ cells/mL were seeded in multiwell plates and, after 24 h, treated according to the experimental design. When HUVECs were incubated with CM_cont or CM_S1, LVES was added to the medium to provide the necessary supplements for endothelial cell growth, which are not present in RPMI1640. When required, infliximab (IFX, 200 µg/mL), baricitinib (1 µM), anti-IFNγ antibody (4 µg/mL), anti-IL-1β antibody (10 µg/mL), and anti-IL-6 antibody (10 µg/mL) were added to CM_S1. In experiments evaluating the effects of cytokines, HUVECs were incubated in RPMI1640 supplemented with LVES; 5 ng/mL of IFNγ or IL-1, or 50 ng/mL of TNFα or IL-6 (R&D Systems by Bio-Techne, Milano, Italy) were added individually or in combination (cytomix).

### 4.2. RT-qPCR Analysis

For gene expression analysis, cells, seeded in 24-well plates, were treated according to the experimental design. At indicated times, the total RNA, extracted with the GeneJET RNA Purification Kit (Thermo Fisher Scientific), was converted to cDNA with the RevertAid First Strand cDNA Synthesis Kit (Thermo Fisher Scientific). Gene expression levels were determined with Real-Time PCR using specific forward and reverse primer pairs (Table 1) and SYBR™ Green Master Mix (Thermo Fisher Scientific); the expression of RPL15 was employed as a loading control. The results are shown as a fold change relative to the control group (=1) calculated with the ∆∆Ct method.

### 4.3. Western Blot Analysis

For the analysis of protein expression, cell lysates were prepared using LDS sample buffer (Thermo Fisher Scientific) and analyzed as already described [26]. Briefly, proteins were separated through electrophoresis on a Bolt™ 4–12% Bis-Tris mini protein gel (Thermo Fisher Scientific) and transferred to PVDF Immobilon-P membranes (Thermo Fisher Scientific). The membranes were then maintained for 1 h at room temperature in TBST (20 mM Tris, 150 mM NaCl, 0.5% Tween 20, pH 7.4) and 4% non-fat dried milk, then overnight at 4 °C in TBST and 5% BSA containing the following antibodies (1:2000, Cell Signaling Technology, Euroclone, Milano, Italy): rabbit polyclonal anti-eNOS, anti-Arginase2, anti-phospho-Akt (Thr308), or anti-phospho-NFκB p65 (Ser536). Anti-vinculin mouse monoclonal antibody (1:2000, Merck) was employed as a loading control. HRP-conjugated secondary antibodies (anti-rabbit and anti-mouse IgG, 1:10,000, Cell Signaling Technology) were then employed to detect protein bands, along with SuperSignal™ West Pico PLUS Chemiluminescent HRP Substrate (Thermo Fisher Scientific). Western blot images were captured using an iBright FL1500 Imaging System (Thermo Fisher Scientific) and analyzed with iBright Analysis Software (version 5.0). 

### 4.4. Arginine Uptake

The transport of arginine was measured in HUVECs seeded in 24-well plates (Falcon, Italy). After the experimental treatments, cells were washed twice with Earle’s Balanced Salt Solution (EBSS), then incubated for 1 min in the same buffer added with 50 µM [^3^H]arginine (4 μCi/mL) with or without leucine or leucine + lysine (2 mM each), used to inhibit System y+L or y+, respectively. The incubation ended with two quick washes (<10 s) with ice-cold 300 mM urea. Intracellular radioactivity from the cell monolayers was extracted using ethanol and quantified with a MicroBeta2^®^ liquid scintillation counter (Perkin Elmer, Milano, Italy). Uptake data were normalized to protein content measured with the Lowry method and expressed as nmol of arginine uptake per mg of protein per minute. System y+L activity was calculated as the fraction of arginine uptake inhibited by leucine, while System y+ activity was determined as the rate further inhibited by lysine [58].

### 4.5. Determination of Intracellular Arginine

The content of intracellular arginine was measured using HPLC, following a previously outlined method [58]. After the experimental treatments, HUVECs, cultured in 6-well plates, were quickly washed with ice-cold PBS, then incubated for 10 min in ice-cold ethanol to extract the intracellular pool of amino acids. After freeze-drying, pellets were resuspended in Lithium Loading Buffer (Biochrom, Cambridge, UK) and loaded on a Biochrom 30 amino acid analyzer (Biochrom) for the separation of amino acids and their quantification with a ninhydrin-based derivatization reaction. Arginine content was expressed as nmol per mg of cell protein.

### 4.6. Cytokine Analysis

The amount of TNFα, IL-1β, IL-6, and IFNγ secreted in the culture medium of Spike S1-activated macrophages (CM_S1) was determined with the Human Quantikine ELISA kits (R&D Systems, Bio-Techne, Milano, Italy), specific for each cytokine, as specified by the manufacturer.

### 4.7. Nitric Oxide Production

The production of nitric oxide (NO) was determined by measuring the amount of nitrites, derivatives of NO, in the culture medium. To this end, the fluorescent molecule 1-(H)-naphtotriazole from 2,3-diaminonaphthalene (DAN) was employed, as previously described [59]. Briefly, 100 μL of the cell medium was mixed with 20 μL of DAN (0.025 mg/mL in 0.31 M HCl). After 10 min, 20 μL of 0.7 M NaOH was added, and fluorescence was read with the EnSpire^®^ Multimode Plate Reader (PerkinElmer, Milano, Italy). Nitrite production was expressed as nmoles/mL of the extracellular medium (μM).

### 4.8. Statistical Analysis

Statistical analyses were performed using GraphPad Prism, version 8.4.2 (GraphPad Software, Boston, MA, USA). *p*-values were determined using Ordinary One-way ANOVA for multiple comparisons or Student’s *t*-test, as indicated in the legend of each figure. A *p*-value of less than 0.05 was considered indicative of statistical significance.

### 4.9. Materials

Recombinant human cytokines were obtained from R&D Systems by Bio-Techne, Milano, Italy. Endotoxin-free fetal bovine serum was sourced from Thermo Fisher Scientific. L-[2,3,4-^3^H]-monohydrochloride Arginine (54.5 mCi/mmol) was supplied by Perkin Elmer. All other chemicals were purchased from Merck (Milano, Italy), unless stated otherwise. The anti-TNFα monoclonal antibody infliximab, IFX (Remsima), was from Celltrion Healthcare Italy (Milano, Italy); anti-hIL-1β, anti-h/prIL-6, and anti-IFNγ antibodies were from R&D Systems.

## 5. Conclusions

Our findings demonstrate that cytokines secreted by macrophages activated by spike S1 of SARS-CoV-2 cause a marked decrease in eNOS along with an increase in arginase and a concomitant increase in intracellular arginine in endothelial cells. This impairment of arginine metabolism may contribute to endothelial dysfunction in COVID-19. The efficacy of IFX and baricitinib in correcting either eNOS or arginase unbalance in vitro can open new fields of investigation for the combined use of these drugs to target endothelial dysfunction in COVID-19.

## Figures and Tables

**Table 1:** Sequences of the primer pairs employed for RT-qPCR analysis.

Gene/Protein	Forward Primer	Reverse Primer
SLC7A1/CAT1	CTTCATCACCGGCTGGAACT	GGGTCTGCCTATCAGCTCGT
SLC7A2/CAT2B	TTCTCTCTGCGCCTTGTCAA	CCATCCTCCGCCATAGCATA
SLC7A6/y+LAT2	TaqMan^®^ Gene Expression Assay (Cat# Hs00187757_m1)	
SLC7A7/y+LAT1	TaqMan^®^ Gene Expression Assay (Cat# Hs00374417_m1)	
NOS3/eNOS	TGGTACATGAGCACTGAGATCG	CCACGTTGATTTCCACTGCTG
ARG2/arginase	AAGCTGGCTTGATGAAAAGGC	GCGTGGATTCACTATCAGGTTGT
RPL15/RPL15	GCAGCCATCAGGTAAGCCAAG	AGCGGACCCTCAGAAGAAAGC

## Data Availability

The data that support the findings of this study are openly available in the Center for Open Science_OSF at https://osf.io/qxsm5/ (accessed on 16 May 2025).
